# Assessing regional performance for the Sustainable Development Goals in Italy

**DOI:** 10.1038/s41598-021-03635-8

**Published:** 2021-12-16

**Authors:** Idiano D’Adamo, Massimo Gastaldi, Cesare Imbriani, Piergiuseppe Morone

**Affiliations:** 1 Department of Computer, Control and Management Engineering, Sapienza University of Rome, Rome, Italy; 2 Department of Industrial Engineering, Information and Economics, University of L’Aquila, L’Aquila, Italy; 3 Department of Law and Economics, UnitelmaSapienza University of Rome, Rome, Italy

**Keywords:** Climate-change policy, Sustainability

## Abstract

The monitoring report on progress towards the Sustainable Development Goals (SDGs) in a global context involves a large number of actors as it represents probably the biggest change that our society is implementing. Actions at all levels, from local, regional and national to the aggregation of multiple countries (e.g. EU 27) are needed to achieve a sustainable future. This work focuses on a national perspective (Italy) where multi-criteria decision analysis (MCDA) is used to measure current performance. A sustainability score for each region is calculated from a set of 175 indicators contained in all 17 SDGs. Additionally, sustainability scores are disaggregated along the three pillars – social (1–5, 10, 16, and 17), environmental (6, 13–15) and economic (7–9, 11, and 12). The results highlight the positive performance of northern regions and, in particular, of Trentino Alto Adige, which ranks first in the two considered scenarios. In addition, the relevance of territorial specificities emerges for which the analysis of individual SDGs shows different leading regions. It is noteworthy to highlight the performance of the environmental sub-group of SDGs in southern regions, in contrast to the social and economic sub-groups. Evidently, policy actions are needed to reduce the long-lasting North/South divide—yet the highlighted heterogeneous sustainability performance along the three dimensions calls for well targeted policy measures necessary to regain competitiveness at a European and global level, without compromising with environmental sustainability.

## Introduction

The pursuit of sustainability goals is a feat shared by many researchers, policymakers, and other stakeholders because it is considered essential to improving the quality of human life while respecting the surrounding environment. At the end of the 2021 G7 summits, a strong commitment emerged to support developing countries to provide them with more, better, and faster funding to achieve the sustainability transition. In the context of science and technology studies, researchers efforts have growingly focused on the identification of policies that can foster such change^1–3^. This transition can be facilitated by acting on global carbon pricing, through either taxation or emissions trading, as it is considered the easiest instrument to negotiate and induce behavioural changes^4^.

However, although the pandemic period has reignited the urgency to combat climate change, previous studies already highlighted the positive link between sustainability and resilience^5^. The Adoption of the 2030 Agenda for Sustainable Development and its Sustainable Development Goals (SDGs), targets and related indicators has further pushed towards the sustainable transition^6,7^.

SDGs can be used to support policymaking and should be pursued holistically together^8^. Yet the same countries seem to prioritize certain SDGs at the expenses of others. “Such cherry-picking defies the integrated and indivisible nature of the SDGs, and could negatively impact overall progress on sustainable development globally”^9,10^. To pursue such holistic approach, specific actions are encouraged: the use of Industry 4.0 technologies, the dissemination of education 4.0 to poorer nations, the collaboration between developed and developing countries and the development of a globalized circular economy^11^. However, the SDGs lack an overarching goal and an effective aggregate indicator that monitors progress toward that goal^12^. The literature highlights the need for an aggregate indicator that assesses as much the contributions associated with each SDGs as the interactions between them. Some authors propose a sustainable well-being index (SWI) linked to the SDGs and a comprehensive dynamic model capable of estimating future effects as a function of implemented policies^13^. Other authors introduce a 5SEnSU model, defining the area of sustainability that is least represented by the SDGs: environmental and economic capital should receive more attention in terms of targets towards the 2030 Agenda^14^. Emphasis in the implementation of the SDGs varies by geographic area and this requires further study on the analysis of local contexts to develop a comparative analysis that highlights existing problems^15,16^.

Furthermore, the literature places a great deal of emphasis also on rankings—the goal being to raise awareness and accountability among countries toward achieving SDGs^17–19^. Analyses show that a country's ranking is highly dependent on the method and indicators chosen and that the methods used so far have the limitation of not considering interconnections^20^. These analyses can be conducted not only to a global level^20^, but also at the local level^21^.

Building on previous studies that assessed which are the European countries that have moved faster towards the SDGs^22^, this study aims to analyse a specific country (Italy), monitoring strengths and weaknesses in pursuing SDGs at regional level and identify at the same time characteristics that bind regions within the same nation. As we believe, this objective is important not only in view of the 2030 Agenda but also in the achievement of the Next Generation EU target (which sees Italy playing a central role, receiving just under a third of the total available budget). This work therefore aims to meet three objectives:

- the definition of a methodology easy replicable in order to assess a sustainability score able to compare territorial performance.
- the measurement of the performance of individual regions towards the SDGs through a sustainability score obtained through a multi-criteria analysis;
- the identification of policies to be implemented to resolve gaps in order to facilitate a sustainability transition.

## Materials and methods

The first objective of this work concerns the definition of a methodology able to measure sustainable performance considering multiple perspectives, with indicators characterized by different units of measurement. In this regard the MCDA methodology is employed, provided that it allows combining indicators with different units of measurement in an appropriate way. The regional ranking obtained through the MCDA methodology leads to the second objective of the study. Finally, the decomposition of the analysis into different levels provides useful indications to illustrate the third objective related to policy implications. Consequently, the MCDA acquires a key role in the proposed study.

MCDA is a methodology well established in the scientific literature as well as in applied contexts, as it allows comparisons among multiple alternatives that may also be conflicting^23^. The MCDA provides for the calculation of a final value for each alternative in which this value is obtained as a product between the relevance of the criteria and the values that the criteria assume for each alternative^24^. The multi-criteria analysis consists of two distinct phases in which values and weights are calculated. The result of MCDA analysis is the calculation of a Sustainability Score (dimensionless), obtained aggregating both values and weights of the criteria.

Indeed, a critical element in this analysis is the availability and acquisition of reliable data^25^. Different indicators have different units of measurement and the great advantage of MCDA is to cancel out such differences. In fact, the normalization of all values in the range 0–1 is obtained by identifying a maximum and a minimum value. The normalization concerns a homogeneous panel of data (relative to each individual indicator), thus making in this process both the size and the differences in numerical magnitude among the different indicators irrelevant. A brief description of the SDGs can be found in the supplementary material.

**Figure 1:**
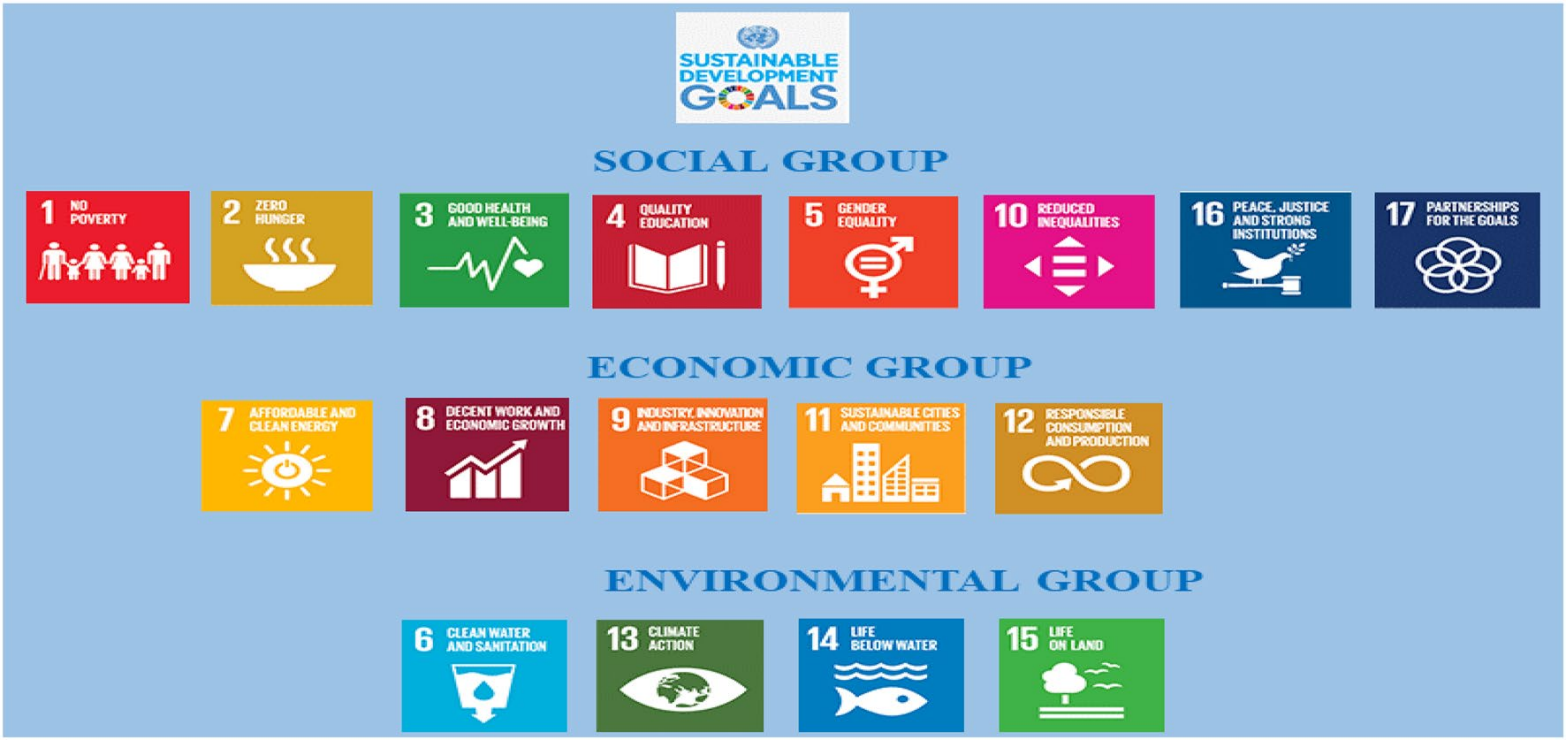
Subdivision of indicators.

The 17 SDGs are clustered into three groups in accordance with existing literature^13,26^ and resembling the three pillars of sustainability: the social group (1–5, 10, 16, and 17), the economic group (7–9, 11, and 12), and the environmental group (6, 13–15)—Fig. 1.

### The identification of input data

The Italian National Institute of Statistics (ISTAT)—ISTAT is engaged in the production of statistical measures to monitor progress towards the SDGs—collects data for each SDG disaggregated at regional level^27^. SDGs are organized into a system of 244 indicators, with which the guidelines for sustainable development are outlined worldwide.

**Figure 2:**
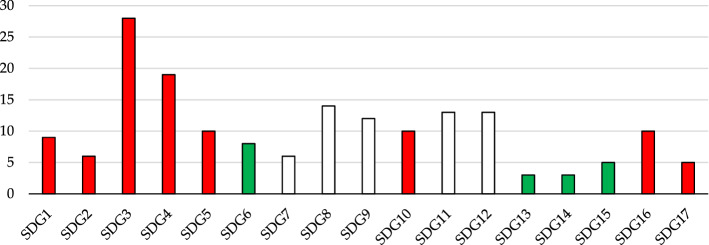
Subdivision of indicators: 19 environmental (green bars), 58 economic (white bars) and 98 socials (red bars).

Building on ISTAT data, we are able to identify 175 indicators (see Supplementary Tables 1–10)—Fig. 2; the remaining 69 indicators are not provided at regional level by ISTAT and therefore are not included in this analysis. Of the available indicators 161 are ready to be used, while 14 need to be normalized according to population. It should be noted that 5 indicators were just doubling information and therefore were removed from our analysis. In the case of missing data, we resorted to other reliable statistical source, whenever available. All data used in this work are available in Supplementary Material.

### Definition of scoring criteria

The assessment of values for each alternative was comprised of 175 input data equal to the number of indicators (criteria). Since the units of measurement were not the same, it was necessary to perform normalization. This approach assigned 1 to the best performing value for each individual alternative (region) and 0 to the least performing^24^. All the regions with an intermediate performance were assigned a value between 0 and 1. It should be mentioned that the desired performance of different indicators bears different slopes, since in some cases an increase is to be preferred (e.g. Households’ satisfaction rate with respect to the continuity of the service of electricity supply), while in others a decrease is to be preferred (e.g. Housing cost overburden rate).

### Definition of weighting factors

The relevance of the criteria is a complex issue since covered sustainability goals are broad in scope and substantially heterogeneous. Hence, it is difficult to identify an objective key for which there is a convergence of thought. Since these are indicators that collectively and holistically points towards sustainability, it is considered inappropriate to prioritise one indicator over another. Bearing this in mind, and considering the need to aggregate the selected set of indicators, the following two methods are proposed:

- Equal weight among SDGs (EWG) scenario.
- Equal weight among indicators (EWI) scenario.

The first considers that all 17 SDGs have the same relevance regardless of the number of indicators that populate the individual SDG. Figure 2 highlights that we go from SDGs 13–14 having 3 indicators to SDG 3 having 28 indicators. Therefore, this scenario has the limitation of penalizing those criteria that are in larger groups. An alternative approach is therefore be to consider all critical indicators regardless of which SDG they belong to.

### Definition of sustainability score

This score is calculated for each alternative, which is represented by the N Italian Regions (IR). Sustainability score is calculated according to the approach proposed in Eqs. (1) and (2) in the EWG scenario and Eqs. (3) and (4) in the EWI scenario:

1 $${\mathrm{Sustainability\,\, Score}}_{\mathrm{IR},\mathrm{SDG}}={\sum }_{\mathrm{I}=1}^{{\mathrm{N}}_{\mathrm{I},\mathrm{SDG}}}{\mathrm{NRV}}_{\mathrm{IR},\mathrm{I}}/{\mathrm{N}}_{\mathrm{I},\mathrm{SDG}}$$

2 $${\mathrm{Sustainability \,\,Score}}_{\mathrm{IR}}={\sum }_{\mathrm{SDG}=1}^{{\mathrm{N}}_{\mathrm{SDG}}}{\mathrm{Sustainability\,\, Score}}_{\mathrm{IR},\mathrm{SDG}}$$

3 $${\mathrm{Sustainability\,\, Value}}_{\mathrm{IR},\mathrm{SDG}}={\sum }_{\mathrm{I}=1}^{{\mathrm{N}}_{\mathrm{I},\mathrm{SDG}}}{\mathrm{NRV}}_{\mathrm{IR},\mathrm{I}}$$

4 $${\mathrm{Sustainability\,\, Score}}_{\mathrm{IR}}={\sum }_{\mathrm{SDG}=1}^{{\mathrm{N}}_{\mathrm{SDG}}}{\mathrm{Sustainability\,\, Value}}_{\mathrm{IR},\mathrm{SDG}}/{\mathrm{N}}_{\mathrm{I},\mathrm{SDG}}$$

in which NRV_IR,I_ is the normalized value of the I indicator for each IR region, N_I,SDG_ is the number of indicators for each SDG and N_SDG_ is the number of SDGs.

In particular, the weight of the 175 indicators will be different in the two scenarios. So if we take one of the 9 indicators associated with SDG1 it has an intermediate weight of 0.111, while it is 0.1667 for one of the 6 indicators in SDG2. These weights are then aggregated across all 17 SDGs, resulting in the overall weights of 0.0065 and 0.0098 within the EWG scenario, respectively. In contrast, in the EWI scenario, the two indicators (taken earlier as examples) have both an overall weight of 0.0057.

In addition, in order to identify any common characteristics, regional data are grouped into three macro-areas:

- North—Valle d’Aosta, Piemonte, Lombardia, Liguria, Trentino Alto Adige, Veneto, Friuli Venezia Giulia and Emilia Romagna.
- Center—Toscana, Umbria, Marche and Lazio.
- South—Abruzzo, Molise, Campania, Puglia, Basilicata, Calabria, Sardegna and Sicilia.

## Results

The results obtained are based on the 3500 observations (175 indicators × 20 regions) and through normalization values were made comparable to each other. With regard to the weights, two different scenarios have been identified and by aggregating all these data, it is possible to obtain a sustainability score for each Italian region.

Supplementary Tables 11–13 present the results for the EWG scenario in which the individual SDGs are normalized in the interval 0–1. Looking at these results at a glance, none of the SDGs reaches its maximum theoretical value of 1; the highest scores reached are 0.96 for Lombardy in SDG13 and 0.95 for Trentino A.A. in SDG1. This occurs because each SDG is composed of multiple indicators and the maximum performance is never achieved in all indicators pertaining to a single SDG.

Supplementary Tables 14–16 show the results of the EWI scenario where individual indicators are aggregated and therefore the maximum value is given by the number of these criteria within the individual SDGs. For example, SDG3 and SDG4 have 28 and 19 indicators respectively, and their maximum value is 19.79 for Liguria and 14.03 for Emilia Romagna. Also in this case the maximum theoretical score is not achieved, since there is no simultaneous maximum performance in all indicators in this two SDGs. In SDG8 Trentino A.A. achieves the highest performance scoring 12.74 in a set of 14 indicators.

**Figure 3:**
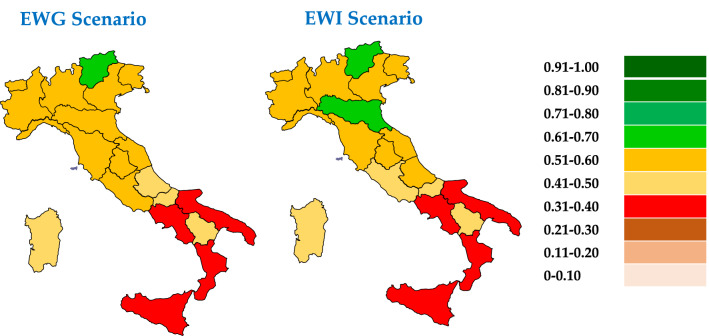
Sustainability Score in both EWG and EWI scenarios—a comparison among Italian regions.

**Figure 4:**
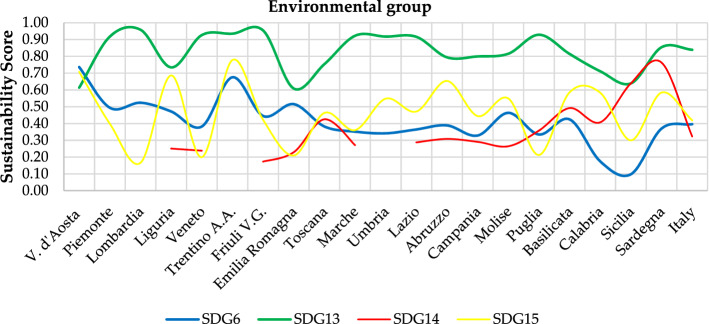
Sustainability Score in EWG scenario—environmental group.

**Figure 5:**
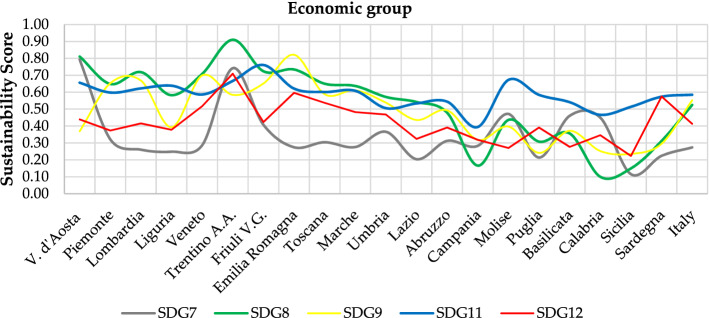
Sustainability Score in EWG scenario—economic group.

**Figure 6:**
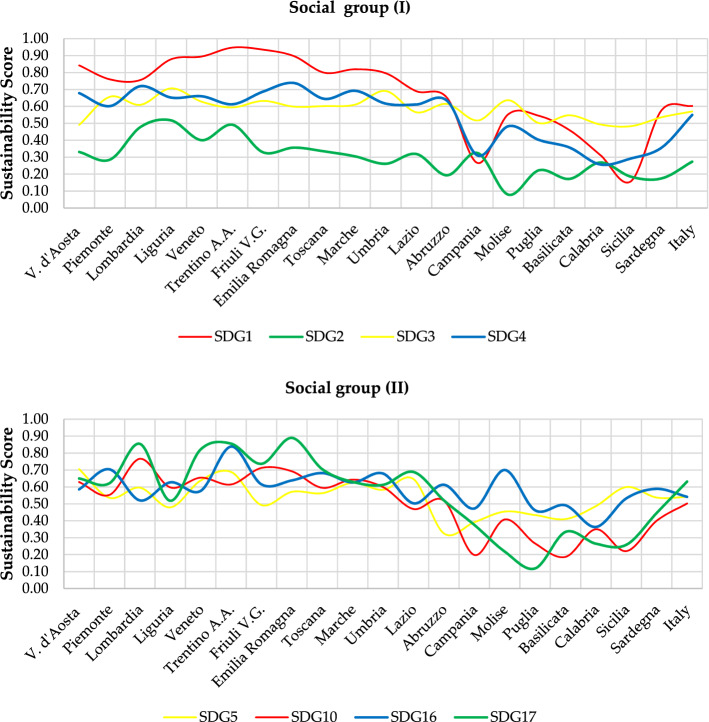
Sustainability Score in EWG scenario—social group.

**Figure 7:**
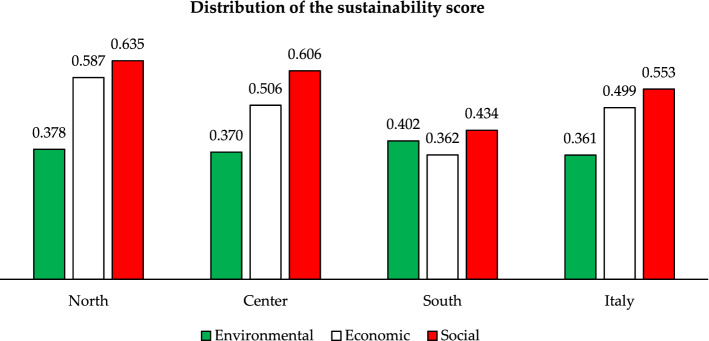
Sustainability Score in EWG scenario—a comparison among macro-areas.

Results can be analysed from different perspectives:

- Regional-level analysis in which the SDGs are aggregated into a synthetic indicator (see Eqs. 2 and 4)—(Fig. 3—Supplementary Table 17).
- The two scenarios are compared by assessing their relative strengths/weaknesses—(Fig. 3—Supplementary Tables 17).
- The Italian regions are compared according to the three groups into which the SDGs have been divided—environmental (Fig. 4), economic (Fig. 5) and social (Fig. 6).
- The three Italian macro-areas are compared according to the three groups of SDGs (Fig. 7—Supplementary Table 18) and the analysis can be further broken down to the level of single SDGs (Supplementary Figs. 1–3).

The sustainability score sees Northern regions excelling, with a leading role played by Trentino in both scenarios with aggregate scores of 0.682 and 0.660. These values, far from the maximum theoretical value of 1, suggest that overall, this region excels but this does not happen in all SDGs. Trentino occupies the first position in SDGs 1, 8, 12, 15 and 16 while for other SDGs the situation is more varied: Liguria outperforms all other regions in SDGs 2 and 3, Emilia Romagna in SDGs 4 and 17, Valle d'Aosta in SDGs 5, 6 and 7, Lombardia in SDG 10, Friuli in SDG 11 and Sardegna in SDG 14. Moreover, depending on the considered scenarios, regions ranking changes (e.g. for SDG 9, Veneto ranks first in the EWG scenario and Emilia Romagna in the EWI scenario; for SDG 13, Friuli Venezia Giulia ranks first in the EWG scenario and Lombardy in the EWI scenario).

Analysing the EWG scenario, the best performing region is Trentino with an overall score of 0.682, followed by Friuli Venezia Giulia and Valle d’Aosta with 0.591. All other Northern and Central regions follow at close distant. The exception is represented by Lazio, which has a value very near to the national average, with 0.502. Below the national average value, we find all Southern regions, led by Abruzzo.

Examining the EWI scenario, the 11 regions above the national average are confirmed, while Lazio assumes a value lower. In addition, we find 15 regions varying their ranking position with respect to the EWG scenario. Under this scenario Trentino is still in the first position with 0.660, while Emilia Romagna scores second, with 0.608. Again, Northern regions, with the exception of Piemonte and Liguria, outperform all Central and Southern regions. Among the regions below the national average (0.505), a Southern region (Abruzzo) comes very close to this average and exceeds Lazio in the ranking.

Similar studies in the literature are not very common. Comparative studies looking at different countries starts to appear in the literature; however, comparison at regional level are still limited. A case in point is the work proposed by Ionescu et al.^28^, whose results are substantially aligned with our findings, showing that Northern regions tend to over perform other regions. Another finding confirmed is that Abruzzo outperforms other Southern regions, while Lazio is the least performing among Central regions.

An additional element to be highlighted is the differences across the two proposed scenarios. The numerical differences of the two scenarios are indeed limited in magnitude. Comparing the first and the last regions differences are in the range of 0.339–0.341 and the single regions vary their performance in a not significant way: 0.022 is the difference marked by Emilia Romagna. While the most negative is associated with Calabria (−0.029).

As discussed, the EWI scenario assigns the same relevance to all indicators, hence reducing possible biases associated with underrepresentation of those indicators pertaining to SDGs with a greater number of indicators (such as SDGs 3, 4, 8 and 11) and overrepresentation of those with a lower number (such as SDGs 13 and 14). However, the EWG scenario has an advantage associated to the normalization process which facilitates comparison, regardless of the number of indicators included in each SDG. Hence, the analysis presented hereafter is based on this latter scenario.

Excluding the regions not bathed by the sea, that present a null value in SDG 14, it is possible to assess and compare the overall performance of individual regions across all 17 SDGs. Among the best performing regions, Trentino Alto Adige does not present specific elements of worries with its lowest score, in SDG 2, at 0.49 (which places it at the second position in the national ranking). Among other Northern regions some common threads emerge in terms of weaknesses in specific SDGs. Poor performances are identified as follows: Emilia Romagna in SDG 7, 14 and 15 (with a range of 0.21–0.27), Friuli Venezia Giulia in SDG 14 (with 0.17), Veneto in SDG 7, 14 and 15 (with a range of 0.20–0.29), Lombardia in SDG 7 and 15 (with a range of 0.17–0.26) and Liguria in SDG 7 and 14 (with 0.25). Piemonte, as mentioned above, occupies a low position in the national ranking, even if its lowest score is 0.29 for SDG 2. Evidently, results above the national average, but yet close to it, impact on the overall performance. A similar situation occurs for several Central regions, like Toscana with its lowest score at 0.30 in SDG 7, while it falls to a lower value for Umbria with SDG 2 (equal to 0.26) and Marche with SDGs 7 and 14 (range 0.27–0.28) and the same for Lazio with 0.20 in SDG 7 and 0.29 in SDG 14. Among Southern regions, Abruzzo has the lowest value in SDG 2 with 0.19.

It is worth noting that given the normalised nature of MCDAs scores, the maximum theoretical value does not necessarily reflect a good performance in absolute terms. In fact, if the ‘best in class’ region scores poorly, this will still get a value of 1 with all other regions ranked below. This leads to two considerations: the first is that it is necessary to monitor the performance of these indicators over time (ISTAT supports this mission), and the second is that where maximum reference values do not reflect a truly good performance, it would be necessary to introduce reduction coefficients in order to highlight poor performances.

Along this normalisation issue, it should be also noted that different indicators have different units of measurement, making differences between the maximum and minimum performance blurry, and cross comparisons among indicators less informative.

More informative are cross-regional comparisons for each SDG. Moreover, comparing regional performances against the national average allows pinpointing elements of weakness across regions. For instance, for SDGs 2 and 7 many regions score below the national average of 0.27 (this being also the lowest value among SDGs) suggesting a large gap between overperforming and underperforming regions and signalling that a large share of regions presents elements of weakness in these SDGs—this being a valuable information for identifying hotspots and defining policy actions. Another interesting finding is related to SDG 13, with the highest national average equal to 0.84 and with a maximum distance to this score of just 0.35—therefore regional performances vary in a narrow range and most regions are located near the best performance side of the spectrum.

Other interesting insights arise when comparing Italian macro regions. Also in this case results refer to the EWG scenario. This analysis will provide a snapshot of macro-regional differences—an issue which has long characterised the Italian economy and has attracted the attention of several scholars in recent and less recent times, e.g.^29,30^.

As far as the comparison of the three macro-areas is concerned, it emerges that the difference between North and Center reaches a maximum of 0.10 in terms of sustainability score for SDG 2 in the social group and SDG 7 in the economic group. The overall result is 0.635 vs 0.606 and 0.587 vs 0.506 in the two groups respectively, both of which are above the national average. The situation changes considerably when comparing North and South, with differences of 0.40 in SDG 1 and 0.30 in SDGs 3, 10 and 17 on the social side and 0.40 in SDG 8 and 0.30 in SDG 9 on the economic side. This reflects in a difference between the sustainability score of the North and the South of 0.225 for the economic SDGs and of 0.202 for the social SDGs. The difference is lower when comparing Central and Southern regions, but remains noteworthy: 0.144 and 0.172 for the economic and social side, respectively. Interestingly, the situation reverts when looking at the sustainability score in the environmental group of the SDGs, which sees the Southern regions overperforming with a score of 0.402 compared to Northern and Central regions reaching 0.378 and 0.370, respectively. These findings reflect the socio-economic divide which has long characterised Italian regions.

The proposed results are in line with findings emerging from a recent ISTAT report, in which northern regions excel (and in particular Trentino Alto Adige), while southern ones suffer. The report shows also that over the period 2010–2019 60.5% of the SDGs related indicators showed an improvement whereas 20.5% showed a worsening. However, when comparint the same indicators over the period 2019–2020 improvements are reduced to 42.5%, while worsening rise to 37.0%^31^.

### Policy implications

The analysis conducted in this paper shows several important patterns arising from the regional comparison which could serve the purpose of drawing relevant policy measures. Specifically, the two proposed scenarios spotlight regions that require urgent attention and rapid policy interventions to realize the 2030 Agenda’s far-reaching vision. Indeed, Southern regions need targeted interventions in order to cover the gap arising specifically in socio-economic related SDGs. However, such intervention should not occur at the expenses of environmental goals, where the relative position of Southern regions is promising. To achieve such challenging target, integrated solutions are needed and a holistic view of the 2030 Agenda should be embraced in order to define well-targeted interventions.

Looking backword can provide valuable insights to design forward-looking policy measure. For two decades beginning in 1951 Southern regions underwent a period of exceptional growth. This trend led to a significant reduction in the output gap between South and North for the first time in the post-unification period. “From 1951 to 1971 per capita GDP in the South rose at an average annual rate of 5.77 per cent, one of the best performances in the entire world. […] The gap in per capita output with the rest of Italy was narrowed from 53 percentage points in 1951 to 44 in 1961 and 33 in 1971. Essentially, the South gained a percentage point of per capita GDP a year, at a time when Italian GDP was growing at historically unequalled rates”^32^.

The convergence process involved all Southern regions and was undoubtedly driven by the massive public policy programs that created the conditions for economic growth in the South. Specifically, we refer to the Southern Italy Development Fund (Cassa per il Mezzogiorno) which was established in 1950 and lasted until 1984. Over the first decade, the Fund was largely dedicated to modernize the agricultural sector creating new infrastructures, developing water systems and modernizing government structure. Subsequently, investments were reoriented towards capital-intensive sectors, directly promoting industrialization^33^. This action was flanked by national programs to strengthen health, welfare and education, which favored the partial socio-economic equalization, as shown by the convergence of the South’s human development indicators with those of the rest of the country^32^.

Building on the successful experience of the first period of the Fund, and taking stocks of the key impact of public investments in infrastructure, health, welfare and education, new policy actions should aim at revamping the anaemic industrial structure of the South both building new green infrastructures as well as new competences for operating in those sector that can best combine economic growth with social and environmental sustainability. These actions would put Southern regions in a position to close the gap with Center and Northern regions, placing themselves in a competitive position on global scale.

In this regard, industrial policies aimed at revamping the economy of Southern regions should be focused on those sectors less impacting on the environment. On this point, the bioeconomy (and more specifically the circular bioeconomy) might well represent a strategic meta-sector for the South of Italy.

Indeed, the key-role of circular bioeconomy towards SDGs is confirmed by a European cross-country comparison^34^. In addition, as noted by Fava et al.^35^ “Italy has the third largest bioeconomy in Europe (€330 billion annual turnover, 2 million employees), making it a core pillar of the national economy”. Moreover, several Southern regions have already a competitive advantage in biobased related sectors including food and biobased products^36^. As a matter of fact, the circular bioeconomy well reconciles economic growth with social and environmental targets, hence prompting improvements along the three pillars of sustainability. “The bioeconomy reduces dependence on fossil fuels and finite materials, loss of biodiversity and changing land use. It contributes to environmental regeneration, spurs economic growth and supports jobs in rural, coastal and abandoned industrial areas, leveraging local contexts and traditions”^35^.

Finally, the need to react to the COVID-19 crisis can be a catalyst for the transition to a more sustainable economy, where well-being is reconsidered through new lenses that must focus on new production and consumption models. This requires structural policies, targeted innovation, access to green finance, risk-taking capacity and sustainable business models. In this sense, the Italian National Recovery and Resilience Plan (PNRR) is well oriented along these strategic lines. It could pave the way to the implementation of a resilient and sustainable model, which places ecological transition at the core of all economic activities achieving, among others, reduction of climate-altering gas emissions, energy efficiency, reforestation, sustainable agriculture, the production of biobased materials, better management of urban and industrial waste and conversion to circularity.

The Recovery and Resilience Plan will cover all 27 EU countries and the potential of the bioeconomy in Europe is significant^23^, as is the contribution of the green economy^37^ and the circular economy^38^. In fact, these three concepts are united by the achievement of the same goal of sustainability^39^.

## Conclusions

Sustainability is a major challenge, not only to provide concrete responses to climate change, but also to support a much-needed socio-economic recovery after the pandemic period. National governments have become fully aware of the severity of the crisis, allocating significant economic resources to recovery plans. Sustainability is not only a goal to be achieved, it must be demonstrated, because only in this way it will be possible to solve current problems. Analyses that achieve these results should be supported and virtuous behaviour rewarded. It is perhaps time for a watershed that favours subsidies "only for truly sustainable actions" and taxes everything that contributes to increased environmental degradation. However, sustainability is not just about carbon taxation, it is also about other serious problems, such as child exploitation, low schooling rates and the severe poverty in which some families live.

In this context, sustainable revolution is a compass to follow because human capital is complementary to natural capital. It is clear that there is a need for a major social change, a collaboration involving countries, different organisations, and citizens themselves to achieve a new model that leads to clean production and responsible consumption. To implement a lifestyle that looks to others and is not only oriented towards a selfish vision. The circular economy rebound has been introduced in the literature as a concept that defines that simply closing material cycles is not enough. However, in this paper we also want to introduce the concept of "green economy rebound": if we produce energy or make products from natural sources, this should not lead people to consume resources when they are not needed for a real need. This action not only causes damage to natural capital but also to human capital, because it means that we are behaving irresponsibly. In fact, it concerns those who only pay attention to what is already there, without paying attention to the needs of future generations. The green economy is a more comprehensive concept than the bioeconomy and the circular economy. Similarly, this new way of producing must be able to meet human needs on a global level. Today this is not the case, as there is too great a gap between industrialised countries and those where people still die of malnutrition. In the same way, inequalities within countries must be overcome.

This paper tries to introduce a method to aggregate the multiple indicators, which tend to be very different from each other. The literature seems to show that the SDGs put more emphasis on the social aspect, which is probably the most difficult to measure among the three pillars of sustainability. The sustainability score proposed in this paper can be easily replicated across countries globally (provided that national statistical offices collect the needed data) and allows for several objectives to be achieved. The regional ranking makes it possible to outline the best performing territories and the breakdown of the data makes it possible to highlight territorial specificities. The results of this work show that Southern Italian regions suffer significantly from a socio-economic point of view, while from an environmental point of view the picture shows a good performance of these very same regions. This work proposes a number of policies to be followed, but it is clear that the non-recovery of Southern regions in Italy cannot only be associated with wrong political choices. It is necessary that the best minds and forces can be trained in these territories, enhancing human capital. These generations must have the opportunity to live in these areas, being able to develop their ideas, creating new business models and acting as a positive example to future generations. In particular, if this part of the country fails to grow, the social differences between the territories will continue and the lack of economic development in this area will be a brake for the whole country. Italy's PNRR, which is strongly based on sustainability and has significant funds for the south of Italy, is the last chance to reclaim a territory with enormous natural potential. Sustainability is the harmony between nature and society, a principle that drives territories to cooperate with each other and to involve citizens in decision-making.

## Supplementary Information

Supplementary Information.
