# Supplementary material for: Assessing regional performance for the Sustainable Development Goals in Italy
Source: Sci Rep. 2021 Dec 16;11:24117. doi: 10.1038/s41598-021-03635-8 (PMC8677844; doi:10.1038/s41598-021-03635-8)
Supplement: Supplementary file 1 — Supplementary Information. [file 41598_2021_3635_MOESM1_ESM.docx]

**Supplementary material**

**Description of Sustainable Development Goals**

SDG1 (No poverty): End all forms of poverty in the world.

SDG2 (Zero hunger): End hunger, achieve food security, improving nutrition and promoting sustainable agriculture.

SDG 3 (Good health and well-being): Ensure health and well-being for all and all ages.

SDG 4 (Quality education): Ensure quality, equitable and inclusive education, and promote lifelong learning opportunities for all.

SDG 5 (Gender equality): Achieve gender equality and empowerment (increased strength, self-esteem, and awareness) of all women and girls.

SDG 6 (Clean water and sanitation): Ensure availability and sustainable management of water and sanitation for all.

SDG 7 (Affordable and clean energy): Ensure access to affordable energy systems for all, reliable, sustainable and modern energy systems.

SDG 8 (Decent work and economic growth): Fostering sustained, inclusive and sustainable economic growth, full and productive employment and decent work for all.

SDG 9 (Industry, innovation and infrastructure): Build a resilient infrastructure and promote innovation and equitable, responsible and sustainable industrialization.

SDG 10 (Reduced inequalities): Reduce inequality within and between nations.

SDG 11 (Sustainable cities and communities): Making cities and human settlements inclusive, safe, durable and sustainable.

SDG 12 (Responsible consumption and production): Ensuring sustainable patterns of production and consumption.

SDG 13 (Climate action): Take urgent action to combat climate change and its consequences.

SDG 14 (Life below water): Conserve and make sustainable use of the oceans, seas and marine resources for sustainable development.

SDG 15 (Life on land): Protect, restore and promote sustainable use of the Earth's ecosystem, sustainably manage forests, combat desertification, halt and reverse land degradation, and halt the loss of biological diversity.

SDG 16 (Peace, justice and strong institutions): Promote peaceful and more inclusive societies for sustainable development; providing access to justice for all and creating effective, accountable and inclusive bodies at all levels.

SDG 17 (Partnership for the goals): Strengthening the means of implementation and renewing the global partnership for sustainable development.

**Remarks for Tables 1-10**

Rows, with symbol (/), indicate that the original values were absolute and have been normalised to population size

Rows, with symbol (&), indicate that the indicators are double

Rows, with symbol (“), indicate that the indicators are repetitive

**Remarks for Tables 11-16**

The symbol * is proposed for SDG14 in some Regions that are not bathed by the sea

**LIST OF TABLES**

Table 1. List of indicators – Part I

Table 2. List of indicators – Part II

Table 3. List of indicators – Part III

Table 4. List of indicators – Part IV

Table 5. List of indicators – Part V

Table 6. List of indicators – Part VI

Table 7. List of indicators – Part VII

Table 8. List of indicators – Part VIII

Table 9. List of indicators – Part IX

Table 10. List of indicators – Part X

Table 11. Sustainability Score for each SDG in EWG scenario – Part I

Table 12. Sustainability Score for each SDG in EWG scenario – Part II

Table 13. Sustainability Score for each SDG in EWG scenario – Part III

Table 14. Sustainability Score for each SDG in EWI scenario – Part I

Table 15. Sustainability Score for each SDG in EWI scenario – Part II

Table 16. Sustainability Score for each SDG in EWI scenario – Part III

Table 17. Sustainability Score in both EWG and EWI scenarios – A comparison among Italian regions

Table 18. Sustainability Score in EWG scenario – A comparison among Italian regions for different SDGs group

**LIST OF FIGURES**

Figure 1. Sustainability Score in EWG scenario – A comparison among Italian regions, SDGs environmental group

Figure 2. Sustainability Score in EWG scenario – A comparison among Italian regions, SDGs economic group

Figure 3. Sustainability Score in EWG scenario – A comparison among Italian regions, SDGs social group

Table 1. List of indicators – Part I

| **Indicators** | **Italy** |
| --- | --- |
| SDG 1.2.2 - Proportion of men, women and children of all ages living in poverty in all its dimensions according to national definitions |  |
| At risk of poverty or social exclusion (Istat, 2018, %) | 27.3 |
| Severe material deprivation rate (Istat, 2018, %) | 8.5 |
| Low work intensity (Istat, 2018,%) | 11.3 |
| People at risk of poverty (Istat, 2018, %) | 20.3 |
| SDG 1.4.1 - Proportion of population living in households with access to basic services |  |
| Housing cost overburden rate (Istat, 2018, %) | 8.2 |
| Households very or little satisfied with the continuity of the service of electricity supply (Istat, 2019, %) - (&) | 93.5 |
| Households per difficulties of links with public transport (Istat, 2019, %) | 33.5 |
| Landfill of waste (Ispra, 2018, %) - (&) | 21.5 |
| Households unsatisfied for the continuity of the water supply service (Istat, 2019, %) | 8.6 |
| Househlods with fixed and/or mobile broadband connection (Istat, 2019, %) | 74.7 |
| People aged 6 and more using their mobile phones at least a few time a year (Istat, 2019, %) - (&) | 91.9 |
| SDG 2.2.2 - Prevalence of malnutrition among children under 5 years of age, by type (wasting and overweight) |  |
| Overweight or obesity among minors from 3 to 17 years of age (Istat, 2018, %) | 25.2 |
| SDG 2.3.1 - Volume of production per labour unit by classes of farming/pastoral/forestry enterprise size |  |
| Production per labour unit of time below 15000 euros of turnover per year (Istat, 2015, euros) | 53,228.000 |
| SDG 2.4.1 - Proportion of agricultural area under productive and sustainable agriculture |  |
| Percentage of utilized agricultural area under organic farming (Istat, 2018, %) | 15.5 |
| Growth rate of organic crops (Sinab, 2018, %) | 2.6 |
| Fertilizers distributed in agriculture (Istat, 2018, kgs per hectare) | 509.8 |
| Plant protection products distribuited in agriculture (Istat, 2018, kgs per hectare) | 12.8 |
| SDG 3.2.1 - Under-five mortality rate |  |
| Under-five mortality rate (Istat, 2018, per 1000) | 3.5 |
| SDG 3.2.2 - Neonatal mortality rate |  |
| Neonatal mortality rate (Istat, 2018, per 1000) | 2.0 |
| SDG 3.3.1 - Number of new HIV infections per 1,000 uninfected population, by sex, age and key populations |  |
| Number of new HIV infections per 100000 population (ISS, 2017) | 5.7 |
| SDG 3.4.1 - Mortality rate attributed to cardiovascular disease, cancer, diabetes or chronic respiratory disease |  |
| Age standardized mortality rate between 30-69 years of age from major causes of death (Istat, 2017 per 100000) | 220.1 |
| Healthy life expectancy at birth (Istat, 2018, number of years) | 58.5 |
| Overweight or obesity rate (Istat, 2019, %) | 44.9 |
| SDG 3.4.2 - Suicide mortality rate |  |
| Age standardized suicide mortality rate (Istat, 2017, per 100000) | 5.9 |

Table 2. List of indicators – Part II

| **Indicators** | **Italy** |
| --- | --- |
| SDG 3.5.2 - Alcohol per capita consumption (aged 15 years and older) within a calendar year in litres of pure alcohol |  |
| Alcohol rate (Istat, 2019, %) | 15.8 |
| SDG 3.6.1 - Death rate due to road traffic injuries |  |
| Standardized death rate due to road traffic injuries (Istat, 2018, per 100000) | 5.3 |
| Number of road traffic injuries (Istat, 2018, n per capita) - (/) | 0.000056 |
| Road accidents serious harmfulness rate (Minister of Health, 2018, per 100000) | 30.8 |
| SDG 3.7.1 - Proportion of women of reproductive age (aged 15-49 years) who have their need for family planning satisfied with modern methods |  |
| Demand for family planning satisfied with modern methods (Istat, 2018, %) | 67.2 |
| SDG 3.7.2 - Adolescent birth rate (aged 10-14 years; aged 15-19 years) per 1,000 women in that age group |  |
| Age-specific fertility rate for 1000 women aged 10-14 (Istat, 2018, per 1000) | 0.0290 |
| Age-specific fertility rate for 1000 women aged 15-19 (Istat, 2018, per 1000) | 20.5 |
| SDG 3.8.1 - Proportion of target population covered by essential health services |  |
| Proportion of deliveries with more than 4 check up visits during pregnancy (Minister of Health, 2018, %) | 85.3 |
| Hospital beds rate in public and private care institutions (Minister of Health, 2017, per 10000) | 31.8 |
| Day-hospital beds in public and private care institutions (Minister of Health, 2017, per 10000) | 3.5 |
| Beds in the residential social-healthcare and social-walfare facilities (Istat, 2017, per 10000) | 68.2 |
| SDG 3.9.3 - Mortality rate attributed to unintentional poisoning |  |
| Mortality standardized rate attributed to unintentional poisoning Istat, 2017, per 100000) | 0.4 |
| SDG 3.a.1 - Age-standardized prevalence of current tobacco use among persons aged 15 years and older |  |
| Standardized proportion of people aged 15 and over who report current smoking (Istat, 2019, %) | 19.0 |
| SDG 3.b.1 - Proportion of the target population covered by all vaccines included in their national programme |  |
| Flu vaccination coverage age 65+ (Minister of Health, 2018, per 100) | 53.1 |
| Pediatric vaccination coverage: polio (Minister of Health, 2018, per 100) | 95.1 |
| Pediatric vaccination coverage: measles (Minister of Health, 2018, per 100) | 93.2 |
| Pediatric vaccination coverage: rubella (Minister of Health, 2018, per 100) | 93.2 |
| SDG 3.c.1 - Health worker density and distribution |  |
| Phisicians (Co.Ge.A.P.S., 2019, per 1000) | 4.0 |
| Nurses and midwives (Co.Ge.A.P.S., 2019, per 1000) | 5.9 |
| Dentists (Co.Ge.A.P.S., 2019, per 1000) | 0.8 |
| Pharmacists (Co.Ge.A.P.S., 2019, per 1000) | 1.1 |

Table 3. List of indicators – Part III

| **Indicators** | **Italy** |
| --- | --- |
| SDG 4.1.1 - Proportion of children and young people: (a) in grades 2/3; (b) at the end of primary; and (c) at the end of lower secondary achieving at least a minimum proficiency level in (i) reading and (ii) mathematics, by sex |  |
| Share of students in grade 8 (third year of lower secondary education) performing below the baseline level of proficiency in literacy competence (Invalsi, 2019, %) | 34.4 |
| Share of students in grade 8 (third year of lower secondary education) performing below the baseline level of proficiency in numerical competence (Invalsi, 2019, %) | 38.7 |
| Share of students in grade 8 (third year of lower secondary education) performing below the baseline level of proficiency in english listening competence (Invalsi, 2019, %) | 40.1 |
| Share of students in grade 8 (third year of lower secondary education) performing below the baseline level of proficiency in english reading competence (Invalsi, 2019, %) | 22.4 |
| Literacy competence not adequate (Invalsi, 2019, %) | 30.4 |
| Numerical competence not adequate (Invalsi, 2019, %) | 37.8 |
| SDG 4.1.2 - Completion rate (primary education, lower secondary education, upper secondary education) |  |
| Early leavers from education and training (Istat, 2018, %) | 13.5 |
| SDG 4.2.2 - Participation rate in organized learning (one year before the official primary entry age), by sex ((Miur, 2018, %) |  |
| Participation rate in organized learning (one year before the official primary entry age), by sex (Miur, 2018, %) | 95.4 |
| SDG 4.3.1 - Participation rate of youth and adults in formal and non-formal education and training in the previous 12 months, by sex |  |
| Partecipation in long-life learning (Istat, 2019, %) | 8.1 |
| Participation rate of youth and adults in formal and non-formal education and training in the previous 12 months, by sex (Istat, 2018, %) | 41.5 |
| Students with disabilities, Pre-primary (Miur,2018, %) | 1.5 |
| Students with disabilities, Primary (Miur,2018, %) | 3.2 |
| Students with disabilities, Lower Secondary (Miur,2018, %) | 3.9 |
| Students with disabilities, Upper Secondary (Miur,2018, %) | 2.3 |
| SDG 4.4.1 - Proportion of youth and adults with information and communications technology (ICT) skills, by type of skill |  |
| People with high level of IT competencies (Istat, 2019, %) | 22.0 |
| SDG 4.6.1 - Proportion of population in a given age group achieving at least a fixed level of proficiency in functional (a) literacy and (b) numeracy skills, by sex |  |
| People having completed tertiary education (Istat, 2019, %) | 27.6 |
| SDG 4.a.1 - Proportion of schools offering basic services, by type of services |  |
| Number of schools with pupils with disabilities by adapted computer workstations, Primary (Istat, 2019, %) | 74.9 |
| Number of schools with pupils with disabilities by adapted computer workstations, Lower Secondary (Istat, 2019, %) | 79.9 |
| Number of schools with pupils with disabilities by adapted computer workstations, Upper Secondary (Istat, 2019, %) | 72.2 |

Table 4. List of indicators – Part IV

| Indicators | Italy |
| --- | --- |
| SDG 5.2.1 - Proportion of ever-partnered women and girls aged 15 years and older subjected to physical, sexual or psychological violence by a current or former intimate partner in the previous 12 months, by form of violence and by age |  |
| Proportion of women subjected to home violence (Istat, 2014, %) | 4.9 |
| Anti-violence centres and refuge houses: rate on women aged 14 and over (Istat, 2017, per 100000) | 1.7 |
| SDG 5.2.2 - Proportion of women and girls aged 15 years and older subjected to sexual violence by persons other than an intimate partner in the previous 12 months, by age and place of occurrence |  |
| Proportion of women aged 16-70 subjected to physical or sexual violence by a man other than intimate partner in the previous 5 years (Istat,2014, %) | 7.7 |
| SDG 5.4.1 - Proportion of time spent on unpaid domestic and care work, by sex, age and location |  |
| Ratio of employment rate for women aged 25-49 with at least one child aged 0-5 to the employment rate of women 25-49 years without children (Istat, 2019, %) | 74.3 |
| Proportion of time spent on unpaid domestic and care work (Istat, 2014, %) | 13.5 |
| SDG 5.5.1 - Proportion of seats held by women in (a)national parliaments and (b) local governments |  |
| Women and political representation in Parliament (2018, %) – (&) | 35.4 |
| Proportion of seats held by women in Regional Counsils (Istat, 2020,%) | 21.1 |
| SDG 5.6.1 - Proportion of women aged 15-49 years who make their own informed decisions regarding sexual relations, contraceptive use and reproductive health care |  |
| Abortion rate of women aged 15-49 (Istat, 2017, %) | 5.8 |
| SDG 5.b.1 - Proportion of individuals who own a mobile telephone, by sex |  |
| People aged 6 and more using their mobile phones at least a few time a year (Istat, 2019, %) – (&) | 91.9 |
| People aged 16-74 who used internet once a week in the last 3 months (Istat, 2019, %) | 73.9 |
| SDG 6.1.1 - Proportion of population using safely managed drinking water services |  |
| Water supplied per capita (Istat,2015, litres per capita per day) | 220.0 |
| Households that don't trust to drink tap water (Istat, 2019, %) | 29.0 |
| Households unsatisfied for the continuity of the water supply service (Istat, 2019, %) | 8.6 |
| SDG 6.3.1 - Proportion of domestic and industrial wastewater flow safely treated |  |
| Urban sewage treatment (Istat, 2015, %) | 59.6 |
| SDG 6.3.2 - Proportion of bodies of water with good ambient water quality |  |
| Coastal bathing waters (Ministry of Health, 2018, %) – (&) | 66.5 |
| Percentage of water bodies that have achieved the objective of ecological quality on the total water bodies of surface waters (rivers and lakes) (Ispra, 2015, %) | 41.7 |
| SDG 6.4.1 - Change in water-use efficiency over time |  |
| Urban water supply network efficiency (Istat, 2015, %) | 58.6 |

Table 5. List of indicators – Part V

| **Indicators** | **Italy** |
| --- | --- |
| SDG 6.4.2 - Level of water stress: freshwater withdrawal as a proportion of available freshwater resources |  |
| Freshwater withdrawal for public water supply (Istat, 2018, Millions of m^3^ per capita) – (/) | 0.000156 |
| SDG 6.6.1 - Change in the extent of water-related ecosystems over time |  |
| Wetlands of International Importance (Ispra, 2018, ha per capita) – (/) | 0.00136 |
| SDG 7.1.1 - Proportion of population with access to electricity |  |
| Households very or fairly satisfied with the continuity of the electricity supply service (Istat, 2019, %) | 93.5 |
| SDG 7.2.1 - Renewable energy share in the total final energy consumption |  |
| Renewable energy share in the gross final energy consumption (GSE, 2018, %) | 18.3 |
| Renewable energy share (transport sector excluded) in the gross final energy consumption (GSE, 2018, %) | 17.4 |
| Renewable energy share in thermal sector excluded in the gross final energy consumption (GSE, 2018, %) | 20.1 |
| Share of electricity from renewable sources in the gross final consumption (GSE, 2018, %) | 34.3 |
| Renewable energy share in transport sector in the gross final energy consumption (GSE, 2018, %) | 6.5 |
| SDG 8.3.1 - Proportion of informal employment in total employment, by sector and sex |  |
| Share of employed person not in regular occupation (Istat, 2017, %) | 13.1 |
| SDG 8.4.2 - Domestic material consumption, domestic material consumption per capita, and domestic material consumption per GDP |  |
| Domestic material consumption per capita (Istat, 2018, ton per capita) – (&) | 8.0 |
| Domestic material consumption per GDP (Istat, 2018, ton/thousand euro) – (&) | 0.3 |
| Domestic material consumption (Istat, 2018, ton) – (“) | 484536.0 |
| SDG 8.5.2 - Unemployment rate, by sex, age and persons with disabilities |  |
| Unemployment rate (Istat, 2019, %) | 10.0 |
| Non-partecipation work rate (Istat, 2019, %) | 18.9 |
| Employment rate 15-64 (Istat, 2019, %) | 59.0 |
| Employment rate 20-64 (Istat, 2019, %) | 63.5 |
| Percentage of employed in the total population (Istat, 2019, %) | 39.0 |
| Involuntary part time work (Istat, 2019, %) | 12.2 |
| Share of employed persons with temporary jobs since at least 5 years (Istat, 2019, %) | 17.1 |
| SDG 8.6.1 - Proportion of youth (aged 15-24 years) not in education, employment or training |  |
| People not in education, employment, or training (NEET), aged 15-24 (Istat, 2019, %) | 18.1 |
| People not in education, employment, or training (NEET), aged 15-29 (Istat, 2019, %) | 22.2 |
| SDG 8.8.1 - Fatal and non-fatal occupational injuries per 100,000 workers, by sex and migrant status |  |
| Incidence rate of fatal occupational injuries or injuries leading to permanent disability (INAIL, 2017, per 10000 employed) | 11.4 |

Table 6. List of indicators – Part VI

| **Indicators** | **Italy** |
| --- | --- |
| SDG 8.10.1 - (a) Number of commercial bank branches per 100,000 adults and (b) number of automated teller machines (ATMs) per 100,000 adults |  |
| Number of branches per 100000 inhabitants (Bank of Italy, 2018) | 42.0 |
| Number of ATM per 100000 inhabitants (Bank of Italy, 2018) | 66.8 |
| Number of bank institutions per 100000 inhabitants (Bank of Italy, 2018) | 0.8 |
| SDG 9.2.1 - Manufacturing value added as a proportion of GDP and per capita |  |
| Manufacturing value added as a proportion of total value added (Istat, 2017, %) | 16.7 |
| SDG 9.2.2 - Manufacturing employment as a proportion of total employment |  |
| Manufacturing employment as a proportion of total employment (Istat, 2017, %) | 15.5 |
| SDG 9.3.1 - Proportion of small-scale industries in total industry value added |  |
| Share of manufacturing value added of small-scale manufacturing enterprises on total manufacturing value added (Istat, 2017, %) | 42.1 |
| SDG 9.3.2 - Proportion of small-scale industries with a loan or line of credit |  |
| Percentage of small scale enterprises with at least one line of credit (Istat, 2018, %) | 51.6 |
| SDG 9.5.1 - Research and development expenditure as a proportion of GDP |  |
| R&D Intensity (Istat, 2018, %) | 1.4 |
| Product and/or process innovative enterprises (per 100 enterprises) (Istat, 2016, %) | 38.1 |
| SDG 9.5.2 - Researchers (in full-time equivalent) per million inhabitants |  |
| Researcher in full time equivalent (Istat, 2017, per 10000) | 23.2 |
| Impact of knowledge workers on employment (Istat, 2019, %) | 17.6 |
| SDG 9.b.1 - Proportion of medium and high-tech industry value added in total value added |  |
| Proportion of medium and high tech industry value added in total value added (Istat, 2017, %) | 32.4 |
| SDG 9.c.1 - Proportion of population covered by a mobile network, by technology |  |
| Households with fixed and/or mobile broadband connection (Istat, 2019, %) | 74.7 |
| Enterprises with at least 10 persons employed with connection to internet by fixed and/or mobile broadband (Istat, 2019, %) | 94.5 |
| Enterprises with at least 10 persons employed with web site or an homepage (Istat, 2019, %) | 72.1 |
| SDG 10.1.1 - Growth rates of household expenditure or income per capita among the bottom 40 per cent of the population and the total population |  |
| Growth rate of household income per capita among the bottom 40 per cent of the population (Istat, 2017, %) | 0.2 |
| Growth rate of household income per capita among the total population (Istat, 2017, %) | 1.6 |
| Disposable income inequality (Istat, 2017, ratio of income shares) | 6.1 |
| Pro capite disposable income (Istat, 2019, euro) | 18902.0 |
| SDG 10.2.1 - Proportion of people living below 50 per cent of median income, by sex, age and persons with disabilities |  |
| People at risk of poverty (Istat, 2018, %) | 20.3 |

Table 7. List of indicators – Part VII

| **Indicators** | **Italy** |
| --- | --- |
| SDG 10.7.2 - Number of countries with migration policies that facilitate orderly, safe, regular and responsible migration and mobility of people |  |
| Non EU citizens holding a long-term residence permit (Istat, 2019, n per capita) – (/) | 0.063 |
| Percentage of non EU citizens holding a long-term residence permit (Istat, 2019, %) | 62.3 |
| New permits (Istat, 2018, n per capita) – (/) | 0.0041 |
| Share of permits issued for political asylum and humanitarian motions (Istat, 2018, %) | 26.8 |
| Number of citizenship acquisitions (Istat, 2018, n per capita) – (/) | 0.001899168 |
| SDG 11.1.1 - Proportion of urban population living in slums, informal settlements or inadequate housing |  |
| Share of total population living in houses with structural or humidity problems (Istat, 2018, %) | 13.2 |
| Overcrowding dwellings (Istat, 2018, %) | 27.8 |
| Noise from neighbours or from street (Istat, 2018, %) | 10.9 |
| SDG 11.2.1 - Proportion of population that has convenient access to public transport, by sex, age and persons with disabilities |  |
| Households without easy links with public transport (Istat, 2019, %) | 33.5 |
| Students who travel to their study place only by public transport (Istat, 2019, %) | 28.5 |
| Persons who travel to work by private transport (Istat, 2019, %) | 74.2 |
| SDG 11.3.1 - Ratio of land consumption rate to population growth rate |  |
| Soil sealing from artificial land cover per capita (Ispra, 2018, m^3^/inhab) | 381.0 |
| Illegal building rate (Cresme 2018, value for 100 authorized constructions) | 18.9 |
| SDG 11.5.1 - Number of deaths, missing persons and directly affected persons attributed to disasters per 100,000 population |  |
| Population at risk of landslides (Ispra, 2017, %) | 2.2 |
| Population at risk of flood (Ispra, 2017, %) – (&) | 10.4 |
| SDG 10.7.2 - Number of countries with migration policies that facilitate orderly, safe, regular and responsible migration and mobility of people |  |
| Non EU citizens holding a long-term residence permit (Istat, 2019, n) | 0.063 |
| Percentage of non EU citizens holding a long-term residence permit (Istat, 2019, %) | 62.3 |
| New permits (Istat, 2018, n) | 0.0041 |
| Share of permits issued for political asylum and humanitarian motions (Istat, 2018, %) | 26.8 |
| Number of citizenship acquisitions (Istat, 2018, n) | 0.001899168 |
| SDG 11.1.1 - Proportion of urban population living in slums, informal settlements or inadequate housing |  |
| Share of total population living in houses with structural or humidity problems (Istat, 2018, %) | 13.2 |
| Overcrowding dwellings (Istat, 2018, %) | 27.8 |
| Noise from neighbours or from street (Istat, 2018, %) | 10.9 |
| SDG 11.2.1 - Proportion of population that has convenient access to public transport, by sex, age and persons with disabilities |  |
| Households without easy links with public transport (Istat, 2019, %) | 33.5 |
| Students who travel to their study place only by public transport (Istat, 2019, %) | 28.5 |
| Persons who travel to work by private transport (Istat, 2019, %) | 74.2 |

Table 8. List of indicators – Part VIII

| **Indicators** | **Italy** |
| --- | --- |
| SDG 11.3.1 - Ratio of land consumption rate to population growth rate |  |
| Soil sealing from artificial land cover per capita (Ispra, 2018, m^3^/inhab) | 381.0 |
| Illegal building rate (Cresme 2018, value for 100 authorized constructions) | 18.9 |
| SDG 11.5.1 - Number of deaths, missing persons and directly affected persons attributed to disasters per 100,000 population |  |
| Population at risk of landslides (Ispra, 2017, %) | 2.2 |
| Population at risk of flood (Ispra, 2017, %) | 10.4 |
| SDG 11.6.1 - Proportion of municipal solid waste collected and managed in controlled facilities out of total municipal waste generated, by cities |  |
| Landfill of waste (Ispra, 2018, %) – (&) | 21.5 |
| SDG 11.6.2 - Annual mean levels of fine particulate matter (e.g. PM2.5 and PM10) in cities (population weighted) |  |
| Urban air quality - PM10 (Istat, 2017, % of quality control units exceeding the annual limit) | 22.0 |
| Urban air quality - Nitrogen dioxide (Istat, 2017, % of quality control units exceeding the annual limit) | 11.9 |
| SDG 11.7.1 - Average share of the built-up area of cities that is open space for public use for all, by sex, age and persons with disabilities |  |
| Incidence of urban green areas on urbanized area of the cities (Istat, 2018, m^2^ per 100 m^2^ of urbanized areas) | 8.9 |
| SDG 11.7.2 - Proportion of persons victim of physical or sexual harassment, by sex, age, disability status and place of occurrence, in the previous 12 months |  |
| Persons aged 14-65 years old victims of at least one form of sexual harassment in the last 12 months (Istat, 2016, %) | 5.1 |
| SDG 12.2.2 - Domestic material consumption, domestic material consumption per capita, and domestic material consumption per GDP |  |
| Domestic material consumption per capita (Istat, 2018, ton per capita) – (&) | 8.0 |
| Domestic material consumption per GDP (Istat, 2018, ton/thousand euro) – (&) | 0.3 |
| Domestic material consumption (Istat, 2018, ton) – (“) | 484536.0 |
| SDG 12.4.2 - (a) Hazardous waste generated per capita; and (b) proportion of hazardous waste treated, by type of treatment |  |
| Amount of Hazardous waste (Ispra, 2017, ton per capita) – (/) | 0.163 |
| Hazardous waste sent to the recovery operations (Ispra, 2017, ton per capita) – (/) | 0.073 |
| Hazardous waste sent to the disposal operations (Ispra, 2017, ton per capita) – (/) | 0.088 |
| SDG 12.5.1 - National recycling rate, tons of material recycled |  |
| Separate collection of municipal waste (Ispra, 2018, ton per capita) – (/) | 0.296 |
| Separate collection of municipal waste (Ispra, 2018, %) | 58.2 |
| SDG 12.6.1 - Number of companies publishing sustainability reports |  |
| Public institutions that adopt forms or social and/or environmental reporting (Istat, 2015, %) | 19.5 |
| Number of organizations/enterprises with EMAS registration (Ispra, 2018, n per capita) – (/) | 0.000016 |

Table 9. List of indicators – Part IX

| **Indicators** | **Italy** |
| --- | --- |
| SDG 12.7.1 - Degree of sustainable public procurement policies and action plan implementation |  |
| Public institutions that purchase goods and/or services by adopting minimum environmental criteria (Istat, 2015, %) | 63.2 |
| SDG 12.b.1 - Implementation of standard accounting tools to monitor the economic and environmental aspects of tourism sustainability |  |
| Impact of tourism on waste (Ispra, 2018, kg/equivalent inhabitant) | 9.1 |
| Tourism intensity index (Istat, 2018, per 1000 inhabitants) | 7090.0 |
| Nights spent in open air establishments, farmhouses and mountain refuges on nights spent in all accomodation establishments (Istat, 2018, %) | 18.9 |
| SDG 13.1.1 - Number of deaths, missing persons and directly affected persons attributed to disasters per 100,000 population |  |
| Population at risk of landslides (Ispra, 2017, %) | 10.4 |
| Population at risk of floods (Ispra, 2017, %) | 2.2 |
| Forest fires impact: Area covered by the fire (Istat, 2018, per 1000 km^2^) | 0.6 |
| SDG 14.5.1 - Coverage of protected areas in relation to marine areas |  |
| Marine protected areas EUAP (MATM, 2013, km^2^ per capita) – (/) | 0.00005 |
| Marine areas included in the Natura 2000 network (MATM, 2019, km^2^ per capita) – (/) | 0.00019 |
| Coastal bathing waters (Ministry of Health, 2018, %) | 66.5 |
| SDG 15.1.1 - Forest area as a proportion of total land area |  |
| Forest area as a proportion of total land area (Istat and FAO, 2015, %) | 30.8 |
| Forest area index (Ispra and FAO, 2015, %) | 36.8 |
| SDG 15.1.2 - Proportion of important sites for terrestrial and freshwater biodiversity that are covered by protected areas, by ecosystem type |  |
| Protected natural areas (Istat and Minestry of Environment, 2017, %) | 21.6 |
| SDG 15.3.1 - Proportion of land that is degraded over total land area |  |
| Soil sealing from artificial land cover (Ispra, 2018, %) | 7.6 |
| Fragmentation of natural and agricultural land (Ispra, 2018, %) | 38.8 |
| SDG 16.1.1 - Number of victims of intentional homicide per 100,000 population, by sex and age |  |
| Homicide (Minister of Interior, 2018, per 100000 inhabitants) | 0.6 |
| Intentional Homicide (Minister of Interior, 2018, per 100000 inhabitants) | 0.5 |
| SDG 16.1.4 - Proportion of population that feel safe walking alone around the area they live |  |
| People aged 14 and over feeling unsafe when walking alone in the dark in the area where they live (Istat, 2018, %) | 60.6 |
| SDG 16.3.2 - Unsentenced detainees as a proportion of overall prison population |  |
| Unsentenced detainees as a proportion of overall prison population (Minister of Justice, 2019, %) | 16.5 |
| Prison density (Istat and Minister of Justice, 2019, number of prisoners for 100 available) | 117.9 |

Table 10. List of indicators – Part X

| **Indicators** | **Italy** |
| --- | --- |
| SDG 16.5.1 - Proportion of persons who had at least one contact with a public official and who paid a bribe to a public official, or were asked for a bribe by those public officials, during the previous 12 months |  |
| Households where at least one component has received requests for money, gifts or favours in exchange of favour or services, in the previous 12 months (Istat, 2016, %) | 1.2 |
| SDG 16.6.2 - Proportion of population satisfied with their last experience of public services |  |
| Trust in juridical system (Istat, 2019, score) | 4.7 |
| Trust in other institutions: police and fire brigade (Istat, 2019, score) | 7.5 |
| Percentage of households who find very difficult to reach some basic services (Istat, 2018, %) | 6.9 |
| SDG 16.7.1 - Proportions of positions in national and local institutions, including (a) the legislatures; (b) the public service; and (c) the judiciary, compared to national distributions, by sex, age, persons with disabilities and population groups |  |
| Women and political representation in Parliament (2018, %) – (&) | 35.4 |
| Youth and political representation in Parliament (2018, %) | 42.2 |
| SDG 17.3.2 - Volume of remittances (in United States dollars) as a proportion of total GDP |  |
| Foreign workers' remittances (Istat and Bank of Italy, 2019, millions of Euros per capita) – (/) | 0.0001 |
| Foreign workers' remittances (Istat and Bank of Italy, 2019, %) – (“) | 100.0 |
| SDG 17.6.2 Fixed internet broadband subscriprtions per 100 inhabitants, by speed |  |
| Households with fixed and/or mobile broadband connection (Istat, 2019, %) | 74.7 |
| Enterprises with at least 10 persons employed with connection to Internet by fixed and/or mobile broadband (Istat, 2019, %) | 94.5 |
| SDG 17.8.1 Proportion of individuals using the Internet |  |
| Individuals aged 6 years and over using the Internet in the last 3 months, per 100 individuals (Istat, 2019, %) | 67.9 |
| Enterprises with at least 10 persons employed with web site or an homepage (Istat, 2019, %) | 72.1 |

Table 11. Sustainability Score for each SDG in EWG scenario – Part I

|  | **V. d'Aosta** | **Piemonte** | **Liguria** | **Lom-**  **bardia** | **Veneto** | **Trentino A.A.** | **Friuli V.G.** |
| --- | --- | --- | --- | --- | --- | --- | --- |
| SDG1 | 0.84 | 0.76 | 0.76 | 0.88 | 0.89 | 0.95 | 0.94 |
| SDG2 | 0.33 | 0.29 | 0.52 | 0.48 | 0.40 | 0.49 | 0.33 |
| SDG3 | 0.49 | 0.66 | 0.71 | 0.61 | 0.63 | 0.59 | 0.63 |
| SDG4 | 0.68 | 0.60 | 0.65 | 0.72 | 0.66 | 0.61 | 0.69 |
| SDG5 | 0.71 | 0.54 | 0.48 | 0.60 | 0.64 | 0.69 | 0.49 |
| SDG6 | 0.79 | 0.32 | 0.25 | 0.26 | 0.29 | 0.74 | 0.41 |
| SDG7 | 0.79 | 0.32 | 0.25 | 0.26 | 0.29 | 0.74 | 0.41 |
| SDG8 | 0.81 | 0.65 | 0.58 | 0.72 | 0.71 | 0.91 | 0.72 |
| SDG9 | 0.37 | 0.65 | 0.39 | 0.67 | 0.70 | 0.58 | 0.65 |
| SDG10 | 0.63 | 0.55 | 0.60 | 0.77 | 0.65 | 0.61 | 0.71 |
| SDG11 | 0.66 | 0.60 | 0.62 | 0.64 | 0.59 | 0.67 | 0.76 |
| SDG12 | 0.44 | 0.37 | 0.38 | 0.42 | 0.52 | 0.71 | 0.42 |
| SDG13 | 0.61 | 0.92 | 0.73 | 0.96 | 0.93 | 0.93 | 0.95 |
| SDG14 | * | * | 0.25 | * | 0.24 | * | 0.17 |
| SDG15 | 0.71 | 0.40 | 0.69 | 0.17 | 0.20 | 0.78 | 0.42 |
| SDG16 | 0.59 | 0.64 | 0.62 | 0.51 | 0.55 | 0.79 | 0.56 |
| SDG17 | 0.65 | 0.62 | 0.52 | 0.85 | 0.82 | 0.86 | 0.74 |

Table 12. Sustainability Score for each SDG in EWG scenario – Part II

|  | **Emilia Romagna** | **Toscana** | **Marche** | **Umbria** | **Lazio** | **Abruzzo** | **Campania** |
| --- | --- | --- | --- | --- | --- | --- | --- |
| SDG1 | 0.90 | 0.80 | 0.82 | 0.80 | 0.69 | 0.65 | 0.27 |
| SDG2 | 0.36 | 0.33 | 0.31 | 0.26 | 0.32 | 0.19 | 0.32 |
| SDG3 | 0.60 | 0.60 | 0.61 | 0.69 | 0.57 | 0.61 | 0.52 |
| SDG4 | 0.74 | 0.64 | 0.69 | 0.62 | 0.61 | 0.63 | 0.31 |
| SDG5 | 0.57 | 0.56 | 0.62 | 0.58 | 0.65 | 0.33 | 0.39 |
| SDG6 | 0.52 | 0.38 | 0.35 | 0.34 | 0.36 | 0.39 | 0.33 |
| SDG7 | 0.27 | 0.30 | 0.28 | 0.37 | 0.20 | 0.31 | 0.28 |
| SDG8 | 0.73 | 0.65 | 0.64 | 0.57 | 0.54 | 0.48 | 0.17 |
| SDG9 | 0.82 | 0.59 | 0.61 | 0.54 | 0.44 | 0.49 | 0.32 |
| SDG10 | 0.69 | 0.59 | 0.64 | 0.60 | 0.47 | 0.52 | 0.20 |
| SDG11 | 0.62 | 0.60 | 0.61 | 0.51 | 0.53 | 0.54 | 0.39 |
| SDG12 | 0.60 | 0.54 | 0.48 | 0.47 | 0.32 | 0.39 | 0.32 |
| SDG13 | 0.61 | 0.75 | 0.92 | 0.92 | 0.92 | 0.79 | 0.80 |
| SDG14 | 0.23 | 0.43 | 0.27 | * | 0.29 | 0.31 | 0.29 |
| SDG15 | 0.21 | 0.46 | 0.36 | 0.55 | 0.47 | 0.65 | 0.44 |
| SDG16 | 0.61 | 0.65 | 0.62 | 0.66 | 0.47 | 0.63 | 0.49 |
| SDG17 | 0.89 | 0.70 | 0.63 | 0.61 | 0.69 | 0.52 | 0.38 |

Table 13. Sustainability Score for each SDG in EWG scenario – Part III

|  | **Molise** | **Puglia** | **Basilicata** | **Calabria** | **Sicilia** | **Sardegna** | **Italy** |
| --- | --- | --- | --- | --- | --- | --- | --- |
| SDG1 | 0.55 | 0.54 | 0.46 | 0.31 | 0.16 | 0.58 | 0.60 |
| SDG2 | 0.08 | 0.22 | 0.17 | 0.27 | 0.18 | 0.17 | 0.27 |
| SDG3 | 0.64 | 0.50 | 0.55 | 0.49 | 0.48 | 0.54 | 0.57 |
| SDG4 | 0.48 | 0.40 | 0.36 | 0.26 | 0.29 | 0.36 | 0.55 |
| SDG5 | 0.45 | 0.43 | 0.41 | 0.49 | 0.60 | 0.54 | 0.54 |
| SDG6 | 0.46 | 0.33 | 0.42 | 0.17 | 0.10 | 0.37 | 0.40 |
| SDG7 | 0.47 | 0.21 | 0.46 | 0.45 | 0.12 | 0.22 | 0.27 |
| SDG8 | 0.44 | 0.31 | 0.36 | 0.10 | 0.15 | 0.31 | 0.52 |
| SDG9 | 0.40 | 0.24 | 0.37 | 0.25 | 0.24 | 0.30 | 0.55 |
| SDG10 | 0.41 | 0.27 | 0.19 | 0.35 | 0.22 | 0.40 | 0.50 |
| SDG11 | 0.67 | 0.58 | 0.54 | 0.47 | 0.51 | 0.57 | 0.59 |
| SDG12 | 0.27 | 0.39 | 0.28 | 0.35 | 0.22 | 0.57 | 0.41 |
| SDG13 | 0.82 | 0.93 | 0.81 | 0.71 | 0.64 | 0.85 | 0.84 |
| SDG14 | 0.26 | 0.36 | 0.49 | 0.41 | 0.64 | 0.76 | 0.32 |
| SDG15 | 0.55 | 0.21 | 0.59 | 0.58 | 0.30 | 0.58 | 0.42 |
| SDG16 | 0.73 | 0.49 | 0.47 | 0.37 | 0.57 | 0.57 | 0.54 |
| SDG17 | 0.22 | 0.12 | 0.33 | 0.26 | 0.26 | 0.45 | 0.63 |

Table 14. Sustainability Score for each SDG in EWI scenario – Part I

|  | **V. d'Aosta** | **Piemonte** | **Liguria** | **Lom-**  **bardia** | **Veneto** | **Trentino A.A.** | **Friuli V.G.** |
| --- | --- | --- | --- | --- | --- | --- | --- |
| SDG1 | 7.58 | 6.84 | 6.80 | 7.91 | 8.05 | 8.53 | 8.42 |
| SDG2 | 1.99 | 1.71 | 3.11 | 2.87 | 2.40 | 2.95 | 1.97 |
| SDG3 | 13.73 | 18.39 | 19.79 | 17.07 | 17.57 | 16.64 | 17.71 |
| SDG4 | 12.89 | 11.43 | 12.39 | 13.67 | 12.54 | 11.64 | 13.06 |
| SDG5 | 7.05 | 5.36 | 4.79 | 5.96 | 6.38 | 6.88 | 4.93 |
| SDG6 | 5.89 | 3.95 | 4.19 | 3.77 | 3.06 | 5.40 | 3.56 |
| SDG7 | 4.76 | 1.93 | 1.49 | 1.56 | 1.72 | 4.45 | 2.45 |
| SDG8 | 11.35 | 9.08 | 8.15 | 10.06 | 9.91 | 12.74 | 10.14 |
| SDG9 | 4.43 | 7.83 | 4.72 | 8.03 | 8.39 | 7.01 | 7.82 |
| SDG10 | 6.29 | 5.53 | 5.97 | 7.66 | 6.55 | 6.15 | 7.12 |
| SDG11 | 8.53 | 7.77 | 8.09 | 8.30 | 7.62 | 8.67 | 9.89 |
| SDG12 | 5.71 | 4.85 | 4.92 | 5.41 | 6.73 | 9.22 | 5.50 |
| SDG13 | 1.84 | 2.75 | 2.20 | 2.87 | 2.78 | 2.80 | 2.86 |
| SDG14 | * | * | 0.75 | * | 0.71 | * | 0.52 |
| SDG15 | 3.54 | 1.99 | 3.43 | 0.83 | 1.00 | 3.89 | 2.11 |
| SDG16 | 6.53 | 7.04 | 6.81 | 5.59 | 6.04 | 8.69 | 6.17 |
| SDG17 | 3.25 | 3.11 | 2.59 | 4.27 | 4.11 | 4.28 | 3.68 |

Table 15. Sustainability Score for each SDG in EWI scenario – Part II

|  | **Emilia Romagna** | **Toscana** | **Marche** | **Umbria** | **Lazio** | **Abruzzo** | **Campania** |
| --- | --- | --- | --- | --- | --- | --- | --- |
| SDG1 | 8.08 | 7.19 | 7.37 | 7.17 | 6.21 | 5.86 | 2.39 |
| SDG2 | 2.14 | 2.01 | 1.83 | 1.57 | 1.91 | 1.16 | 1.95 |
| SDG3 | 16.76 | 16.86 | 17.09 | 19.35 | 15.83 | 17.21 | 14.47 |
| SDG4 | 14.03 | 12.25 | 13.15 | 11.71 | 11.62 | 12.04 | 5.89 |
| SDG5 | 5.71 | 5.63 | 6.24 | 5.83 | 6.46 | 3.26 | 3.91 |
| SDG6 | 4.12 | 3.06 | 2.81 | 2.73 | 2.92 | 3.11 | 2.63 |
| SDG7 | 1.65 | 1.83 | 1.66 | 2.20 | 1.22 | 1.87 | 1.70 |
| SDG8 | 10.28 | 9.10 | 8.92 | 8.01 | 7.59 | 6.70 | 2.32 |
| SDG9 | 9.86 | 7.06 | 7.34 | 6.46 | 5.23 | 5.91 | 3.82 |
| SDG10 | 6.92 | 5.95 | 6.43 | 5.99 | 4.69 | 5.19 | 1.98 |
| SDG11 | 8.08 | 7.82 | 7.91 | 6.57 | 6.94 | 7.07 | 5.13 |
| SDG12 | 7.75 | 6.98 | 6.27 | 6.09 | 4.21 | 5.08 | 4.14 |
| SDG13 | 1.83 | 2.26 | 2.77 | 2.75 | 2.75 | 2.38 | 2.40 |
| SDG14 | 0.69 | 1.28 | 0.81 | * | 0.86 | 0.93 | 0.87 |
| SDG15 | 1.05 | 2.32 | 1.80 | 2.74 | 2.36 | 3.27 | 2.22 |
| SDG16 | 6.74 | 7.18 | 6.80 | 7.26 | 5.20 | 6.88 | 5.34 |
| SDG17 | 4.45 | 3.52 | 3.15 | 3.06 | 3.44 | 2.60 | 1.88 |

Table 16. Sustainability Score for each SDG in EWI scenario – Part III

|  | **Molise** | **Puglia** | **Basilicata** | **Calabria** | **Sicilia** | **Sardegna** | **Italy** |
| --- | --- | --- | --- | --- | --- | --- | --- |
| SDG1 | 4.98 | 4.90 | 4.14 | 2.82 | 1.42 | 5.19 | 6.03 |
| SDG2 | 0.47 | 1.34 | 1.03 | 1.61 | 1.11 | 1.05 | 1.64 |
| SDG3 | 17.83 | 14.05 | 15.33 | 13.82 | 13.53 | 15.00 | 15.97 |
| SDG4 | 9.17 | 7.64 | 6.78 | 4.89 | 5.56 | 6.77 | 10.45 |
| SDG5 | 4.54 | 4.34 | 4.10 | 4.87 | 5.99 | 5.37 | 5.41 |
| SDG6 | 3.71 | 2.68 | 3.40 | 1.40 | 0.78 | 2.96 | 3.17 |
| SDG7 | 2.83 | 1.28 | 2.76 | 2.70 | 0.69 | 1.34 | 1.64 |
| SDG8 | 6.11 | 4.31 | 4.99 | 1.40 | 2.09 | 4.36 | 7.35 |
| SDG9 | 4.76 | 2.90 | 4.46 | 3.03 | 2.82 | 3.54 | 6.63 |
| SDG10 | 4.08 | 2.66 | 1.87 | 3.50 | 2.21 | 4.00 | 5.02 |
| SDG11 | 8.74 | 7.60 | 7.03 | 6.06 | 6.68 | 7.46 | 7.61 |
| SDG12 | 3.51 | 5.09 | 3.60 | 4.50 | 2.91 | 7.47 | 5.37 |
| SDG13 | 2.45 | 2.79 | 2.44 | 2.13 | 1.92 | 2.56 | 2.52 |
| SDG14 | 0.79 | 1.07 | 1.48 | 1.22 | 1.92 | 2.29 | 0.97 |
| SDG15 | 2.75 | 1.06 | 2.93 | 2.92 | 1.50 | 2.92 | 2.09 |
| SDG16 | 8.00 | 5.37 | 5.21 | 4.04 | 6.29 | 6.23 | 6.50 |
| SDG17 | 1.09 | 0.59 | 1.67 | 1.32 | 1.29 | 2.23 | 3.16 |

Table 17. Sustainability Score in both EWG and EWI scenarios – A comparison among Italian regions

| **Ranking** | **Region** | **Sustainability Score** | **Ranking** | **Region** | **Sustainability Score** |
| --- | --- | --- | --- | --- | --- |
| 1 | Trentino A. A. | 0.682 | 1 | Trentino A.A. | 0.660 |
| 2 | Friuli V.G. | 0.591 | 2 | Emilia Romagna | 0.608 |
| 3 | V. d'Aosta | 0.591 | 3 | Friuli V.G. | 0.599 |
| 4 | Emilia Romagna | 0.586 | 4 | Veneto | 0.587 |
| 5 | Veneto | 0.576 | 5 | Lombardia | 0.587 |
| 6 | Lombardia | 0.571 | 6 | Valle d'Aosta | 0.578 |
| 7 | Toscana | 0.565 | 7 | Marche | 0.570 |
| 8 | Marche | 0.558 | 8 | Toscana | 0.568 |
| 9 | Liguria | 0.545 | 9 | Liguria | 0.559 |
| 10 | Umbria | 0.534 | 10 | Piemonte | 0.553 |
| 11 | Piemonte | 0.533 | 11 | Umbria | 0.549 |
| 12 | Lazio | 0.502 |  | **Italy** | **0.505** |
|  | **Italy** | **0.502** | 12 | Abruzzo | 0.502 |
| 13 | Abruzzo | 0.497 | 13 | Lazio | 0.493 |
| 14 | Sardegna | 0.479 | 14 | Molise | 0.473 |
| 15 | Molise | 0.465 | 15 | Sardegna | 0.438 |
| 16 | Basilicata | 0.427 | 16 | Basilicata | 0.406 |
| 17 | Puglia | 0.385 | 17 | Puglia | 0.375 |
| 18 | Calabria | 0.370 | 18 | Campania | 0.344 |
| 19 | Campania | 0.365 | 19 | Calabria | 0.341 |
| 20 | Sicilia | 0.334 | 20 | Sicilia | 0.314 |

Table 18. Sustainability Score in EWG scenario – A comparison among Italian regions for different SDGs group

| **Region** | **Environmental group** | **Economic group** | **Social group** |
| --- | --- | --- | --- |
| V. d'Aosta | 0.507 | 0.590 | 0.599 |
| Piemonte | 0.334 | 0.533 | 0.600 |
| Liguria | 0.394 | 0.464 | 0.629 |
| Lombardia | 0.263 | 0.565 | 0.656 |
| Veneto | 0.310 | 0.583 | 0.643 |
| Trentino A.A. | 0.557 | 0.713 | 0.664 |
| Friuli V.G. | 0.397 | 0.607 | 0.637 |
| Emilia Romagna | 0.260 | 0.638 | 0.655 |
| **North macro-area** | **0.378** | **0.587** | **0.635** |
| Toscana | 0.384 | 0.556 | 0.612 |
| Marche | 0.352 | 0.544 | 0.627 |
| Umbria | 0.384 | 0.497 | 0.626 |
| Lazio | 0.360 | 0.427 | 0.559 |
| **Center macro-area** | **0.370** | **0.506** | **0.606** |
| Abruzzo | 0.422 | 0.452 | 0.547 |
| Campania | 0.360 | 0.290 | 0.382 |
| Molise | 0.441 | 0.440 | 0.507 |
| Puglia | 0.310 | 0.359 | 0.413 |
| Basilicata | 0.481 | 0.387 | 0.405 |
| Calabria | 0.448 | 0.300 | 0.372 |
| Sicilia | 0.301 | 0.258 | 0.378 |
| Sardegna | 0.456 | 0.410 | 0.463 |
| **South macro-area** | **0.402** | **0.362** | **0.434** |
| **Italy** | **0.507** | **0.499** | **0.553** |

Figure 1. Sustainability Score in EWG scenario – A comparison among Italian regions, SDGs environmental group

Figure 2. Sustainability Score in EWG scenario – A comparison among Italian regions, SDGs economic group

Figure 3. Sustainability Score in EWG scenario – A comparison among Italian regions, SDGs social group
